# Antimicrobial and Antiproliferative Coatings for Stents in Veterinary Medicine—State of the Art and Perspectives

**DOI:** 10.3390/ma16216834

**Published:** 2023-10-24

**Authors:** Szymon Graczyk, Robert Pasławski, Arkadiusz Grzeczka, Urszula Pasławska, Beata Świeczko-Żurek, Klaudia Malisz, Ketul Popat, Alina Sionkowska, Patrycja Golińska, Mahendra Rai

**Affiliations:** 1 Institute of Veterinary Medicine, Department of Biological and Veterinary Sciences, Nicolaus Copernicus University, Lwowska 1, 87-100 Torun, Poland; robert.paslawski@umk.pl (R.P.); grzeczka@umk.pl (A.G.); urszula.paslawska@umk.pl (U.P.); 2 Department of Biomaterials Technology, Faculty of Mechanical Engineering and Ship Technology, Gdansk University of Technology, Gabriela Narutowicza 11/12, 80-229 Gdansk, Poland; beata.swieczko-zurek@pg.edu.pl (B.Ś.-Ż.); klaudia.malisz@pg.edu.pl (K.M.); 3 Department of Mechanical Engineering, Colorado State University, Fort Collins, CO 80523, USA; ketul.popat@colostate.edu; 4 Department of Biomaterials and Cosmetic Chemistry, Faculty of Chemistry, Nicolaus Copernicus University in Torun, Gagarina 7, 87-100 Torun, Poland; 5 Department of Microbiology, Nicolaus Copernicus University, ul. Lwowska 1, 87-100 Torun, Poland; golinska@umk.pl; 6 Department of Chemistry, Federal University of Piaui (UFPI), Teresina 64049-550, Brazil; mahendrarai7@gmail.com

**Keywords:** stent, tracheal collapse, ureter obstruction, stent modification, biofilm formation, veterinary medicine

## Abstract

Microbial colonization in veterinary stents poses a significant and concerning issue in veterinary medicine. Over time, these pathogens, particularly bacteria, can colonize the stent surfaces, leading to various complications. Two weeks following the stent insertion procedure, the colonization becomes observable, with the aggressiveness of bacterial growth directly correlating with the duration of stent placement. Such microbial colonization can result in infections and inflammations, compromising the stent’s efficacy and, subsequently, the animal patient’s overall well-being. Managing and mitigating the impact of these pathogens on veterinary stents is a crucial challenge that veterinarians and researchers are actively addressing to ensure the successful treatment and recovery of their animal patients. In addition, irritation of the tissue in the form of an inserted stent can lead to overgrowth of granulation tissue, leading to the closure of the stent lumen, as is most often the case in the trachea. Such serious complications after stent placement require improvements in the procedures used to date. In this review, antibacterial or antibiofilm strategies for several stents used in veterinary medicine have been discussed based on the current literature and the perspectives have been drawn. Various coating strategies such as coating with hydrogel, antibiotic, or other antimicrobial agents have been reviewed.

## 1. Introduction

There are several organ systems in the mammalian body that have tubular components in their structure. Often, in the course of certain pathologies, their various constrictions or obstructions occur. Such flow inactivation in human medicine most often affects the urinary system, especially the ureters, the coronary arteries, as well as the respiratory system [1,2,3]. All these lead to severe disorders related to the proper drainage of urine, perfusion of the cardiac tissue, or gas exchange in the lungs [4]. Systemic diseases, such as respiratory, cardiac, or renal failure can be triggered by situations related to constriction of the draining as well as supplying structures. These include unilateral or bilateral obstruction of the ureters, stenosis of certain vessels leading to stroke, as well as stenosis of the trachea and/or bronchi [5,6]. Such situations significantly impair organ function resulting in clinical deterioration, in some cases leading to the death of the patient. For this reason, procedures to ensure the patency of the organ in question were contemplated, presenting the design of a stent for a dog for the first time in 1954 [7]. A stent refers to a tubular structure capable of supporting the physiological shape of the feeding as well as draining structure of a given organ ensuring its patency. The choice of the material from which the stent is made very often depends on the condition of the patient, as well as the availability of the stent in question, but the most common designs we distinguish are based on metal as well as polymeric varieties of stents. Due to its high biocompatibility as well as shape memory, the most commonly used type of stent is the nitinol stent, which is a combination of nickel and titanium alloy [8]. Stenting procedures have become a rapidly growing technique used as an ambulatory, often life-saving procedure in veterinary medicine, although on a much smaller scale than human medicine. Stenting procedures in veterinary medicine mostly involve the respiratory system (trachea, bronchi) and urinary system [9]. The most common indication for stent implantation in dogs and cats is tracheal collapse, as it is a common and vexing clinical problem mainly affecting a large number of small and miniature breed dogs [10,11]. With the more frequent use of stents, an alarming increase in the number of short-, medium-, and long-term postoperative complications has been noted. Typically, respiratory tract stenting leads to restenosis, which is the re-narrowing of the lumen of a given structure in front of, behind, or in the area of the stent [9]. Urinary tract stenting is also associated with postoperative complications, but these are most often related to nosocomial infections and biofilm formation in the lumen of the stent [12]. Biofilm cannot be removed by conventional methods, forcing surgeons to reoperate and remove or possibly replace the stent. In addition, the reported worsening antibiotic resistance around the world and the associated regulations prohibiting the use of more and more antibiotics in veterinary medicine pose increasingly difficult antimicrobial challenges [13]. The research conducted on the ureter shows that additionally, the prophylactic use of antibiotics prior to surgery rarely prevents biofilm formation in the ureter and the development of bacterial infections [14,15]. To reduce these complications in human medicine, it has been decided to coat stents with drugs, polymers, antimicrobial peptides, or nanoparticle coatings that reduce these complications. Preclinical in vitro studies in many cases have shown inhibition of the growth of selected bacterial strains as well as biofilm formation. This was confirmed in some cases by in vivo studies, where the laboratory animal models were mainly mice or rabbits, and in some cases, healthy dogs. Regarding the significant postoperative complications associated with tracheal stenting, current research is focused not only on creating a stent with antimicrobial properties but also on the reduction of granuloma formation [16,17]. These solutions are related to using cytostatic drugs such as paclitaxel or cisplatin, as well as immunomodulatory agents that include sirolimus. In veterinary medicine, the use of such solutions was mainly scientific or performed as a necessary research step in the registration process of a new generation of stents. Despite many innovative resolutions, in veterinary medicine, stenting procedures still result in postoperative complications [18,19].

Therefore, the purpose of this review is as follows:

(1) To focus on the knowledge of stenting in veterinary medicine for the most common diseases;
(2) To present the knowledge gathered so far on the applications of modified stents in veterinary medicine;
(3) To describe the possible applications of antimicrobial and antiproliferative coatings and their possible use in veterinary medicine;
(4) To determine the direction of future research in the context of stenting procedures and their clinical use in veterinary medicine.

## 2. Stents in Veterinary Medicine

### 2.1. Indications for Stenting in Veterinary Medicine

**Figure 1:**
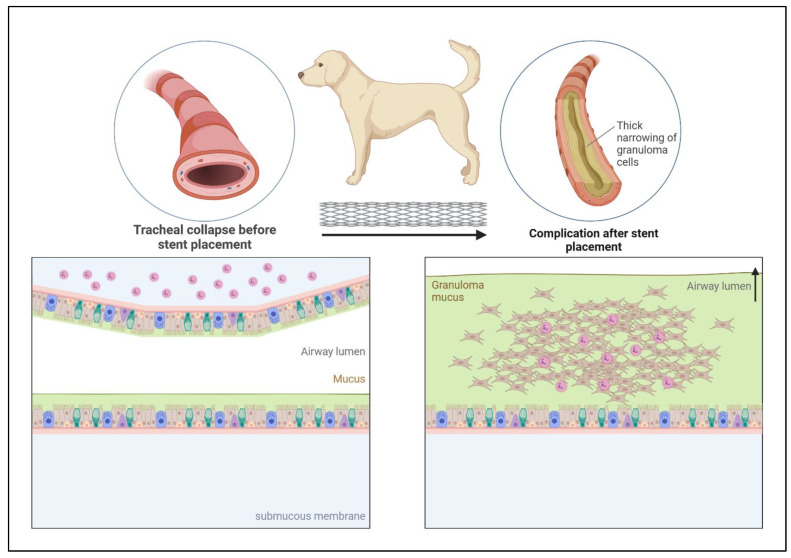
Hyperproliferation of granulation tissue, associated with inflammation after tracheal stent placement in the dog’s trachea with tracheal collapse.

Stenting in veterinary medicine has become a well-known procedure, but is not as frequently used as in humans. Initially, it was thought that the procedure would be a first-line treatment for conditions related to stenosis of the exit routes of various organs in the animal body, such as the trachea and ureters. However, currently, scientific papers, as well as clinical practice, show a significant number of postoperative complications in the short, medium, as well as long term, not always improving the quality of life of the animal [18,19,20,21,22,23,24,25,26,27,28,29,30,31,32]. Respiratory tract collapses are conventionally treated using pharmacological agents—the first-line treatment, and stent placement seems to be one of the definitive approaches [33]. One of the reasons for such late implantation is the constant irritation of the trachea after stent placement initiated by the organism due to the presence of a foreign body [28]. This leads to continuous mechanical stimulation of cough receptors present mainly in the larynx, trachea, and main bronchi. Clinically, it presents as a persistent cough, which is particularly uncomfortable for the animal as well as the owner [34,35]. The second most important complication is the hyperproliferation of granulation tissue overgrowing the stent, leading to re-obstruction of the tracheal lumen (Figure 1). Exaggerated proliferation over time secondarily predisposes to bacterial infections, accelerating the process of restenosis [19,28,31]. Growing granulation tissue has been shown to result from high myofibroblast activity and overproduction of collagen in subepithelial tissue [36]. Bacterial infections themselves after stent placement are not a significant problem because no significant differences have been shown in the formation of bacterial cultures from swabs obtained from the trachea before and after the procedure [37]. Despite the above-mentioned complications, there are situations when endotracheal stent placement is the only solution. One of these is progressive tracheal collapse that does not respond to pharmacological treatment [38]. It is believed that the right time to place such a stent is in progressive tracheal collapse when the tracheal lumen is reduced to 10–20% air permeability. Such deformation significantly impairs breathing clinically manifested by cyanosis, dyspnea, and what is commonly called “goose cough” [32,39]. Therefore, it is believed that the trachea in companion animals (mainly dogs) is the most common site for stent placement, especially in small and miniature breeds, especially Yorkshire Terriers, Pomeranians, or Miniature poodles [24,38,40,41]. In addition to companion animals, cases of tracheal collapse have also been reported in horses [42].

The second most common site predisposing to stent placement, in terms of obstruction, is the urinary exit tract, i.e., the ureters and urethra. This is also the place where we experience adverse complications after stent implantation. The main indication for the insertion of a stent into the ureter is all types of urinary stones that cause their obstruction, where the stent in this case allows urine to drain into the bladder. The most significant postoperative complications are those in the long term. The most commonly mentioned are urinary tract infections [18] caused mainly by *Enterococcus* spp. or *Escherichia coli* [43] which are typical of uropathogenic bacterial cultures [15]. Another equally serious complication is the hyperproliferation of epithelial tissue at the level of the ureterovesical junction, thus leading to restenosis, which results in urine backflow into the kidney causing ureter dilatation [44]. Much less commonly performed, urethral stenting is most often categorized as a palliative treatment for proliferating tumors that, by their mass, press on the urethral wall causing narrowing and often complete occlusion of the urethra. The most commonly mentioned are transitional cell carcinoma, prostatic carcinoma, hemangiosarcoma, or leiomyoma [23,45,46]. In addition to these, there are also non-malignant urethral strictures requiring surgical intervention. Stenosis caused by previous trauma, reflex dyssynergia, urethral epithelial hyperproliferation, or chronic mucosal oedema qualifies for stent placement [46,47]. For this reason, urethral stenting for malignant as well as non-malignant causes is intended to relieve the animal’s pain and allow micturition. However, postoperative complications also occur in this case. In the case of proliferating tumors, the urethral lumen can quickly reseal due to the compressive mass [23]. In addition, urinary incontinence secondary to stent placement—urinary tract infections—and migration or fracture of the stent are observed [23,45,47]. Besides the above-mentioned circumstances, the most common stenting procedures in veterinary medicine are also used in the cardiovascular system [48,49,50,51,52], gastrointestinal system [53,54], or reproductive system [55].

### 2.2. Comparison of Stenting in Human and Veterinary Medicine

Primarily, in human medicine, stents are used much more often than in veterinary medicine. The second difference concerns other diseases that are the most common indication for the procedures: the bile ducts, trachea, the urinary system, and the cardiovascular system, particularly the coronary arteries. In the case of the latter, it is caused by atherosclerosis of the blood vessels due to the deposition of atherosclerotic plaques. Plaques are a combination of lipids from the breakdown of macrophages and also a fibrous component that gradually closes the lumen of the vessel thereby impairing blood flow [56]. In pets, no case of atherosclerosis under physiological conditions has been reported so far. Only those animals fed a high-fat diet classified as hyperresponders who between 1 and 3 months developed severe atherosclerosis after reaching plasma lipoprotein concentrations of 750 mg/100 mL or more, while dogs classified as hyporesponders received the same diet for up to a year without developing vascular atherosclerosis while maintaining plasma lipoprotein concentrations below 750 mg/100 mL [57]. Similarly, in cats, atherosclerosis developed only when a high-fat diet was introduced after at least 4 months, which is unlikely under natural conditions [58]. This suggests that responsiveness to a high-fat diet in these animals is an individual trait. As commercial pet foods are balanced, this problem is not present in veterinary medicine. Due to the high demand for stenting in human medicine and the postoperative complications that do not infrequently arise, mainly related to the overproliferation of the neointima and deposition of red blood cells forming blood clots. It was decided to improve the stents in use by using new stent materials with altered biochemical and biomechanical properties as well as coatings consisting of several compounds ranging from peptides to anticoagulant compounds [59,60,61]. Such solutions are not necessary in veterinary medicine, mainly because there is no need for stents to maintain vascular patency, as no cases of atherosclerosis have been reported in companion animals under physiological conditions. In addition to a well-balanced diet in these animals, age also plays a key role in the progression of this disease, which takes years to develop. Due to the much shorter lifespan of dogs or cats compared to humans, the formation of atherosclerotic plaques in these animals is highly improbable. Nonetheless, there have been cases described in the literature of stent formation in the circulatory system. These included the stenting of pulmonary artery stenosis or an incomplete connection of the odd vein to the caudal vena cava [49,50]. For this reason, knowledge about the use of stents in the cardiovascular system is extremely important, especially when their placement is the only alternative in companion animals. Therefore, knowledge of these procedures and solutions in human medicine is crucial to understanding the procedures themselves and the possible complications associated with the postoperative period in order to choose the best treatment strategy.

### 2.3. Types of Stents and Their Coatings Used in Veterinary Medicine

While human medicine seeks to improve stents based on higher biocompatibility, biodegradability, and fewer post-surgical complications, any changes in veterinary medicine are mainly based on changing the material from which the stent is braided or changing the characteristics of the stent’s braid [32] as well as its insertion system [62], but without bioactive or biodegradable properties [27,29,63,64,65]. The most common material used in the case of a collapsed trachea is nitinol, commercially used for this type of condition, but with a significant number of complications as described above [9]. In addition to nitinol stents based on cross braid, the use of a material called elgiloy has also been described, but with similar effects to the above [31,66]. The recently described nitinol Fauna cross-hook braided stent, due to differences in the arrangement of the nitinol wires, has gained three features that can significantly reduce the number of complications: (1) greater resistance to pressure and tensile forces which reduce the likelihood of migration and shortening of the stent, (2) adaptation of the shape to the trachea under bending, shearing, and torsional forces of the neck which is very valuable in mobile dogs, (3) preservation of the shape of the cells so that their natural structure remains unchanged [32]. In the above-mentioned study, most of the clinical signs before stent placement regressed, except for mild cough in some individuals. However, the study did not mention the important clinical outcome of exaggerated overgrowth of the stent by the tracheal epithelium leading to restenosis, but it is possible that by using a different braiding format, the cells were relieved and thus the problem with excessive epithelial cell proliferation was resolved [32]. Similar short- and medium-term conclusions were documented in a single case of tracheal collapse in a 12-year-old cat [67]. In previous years, it was decided to use a braided Palmaz-type expandable balloon, but it was not successful, showing a high number of complications after its placement [68]. In certain studies, a novel type of stent differs significantly from those previously used. A nitinol spiral stent which was admittedly developed for humans was, however, used in two studies where the experimental group was dogs with tracheal collapse [62,69]. The stent design was characterized by a spirally twisted nitinol wire that was sewn in from the outside with a minimal cut wound of the skin sheath. After the stent was implanted, the cranial, as well as the caudal end of the stent, was sutured to the soft tissues to stabilize the stent in the trachea. This allowed the stent to adapt to the dynamic movements of the head, reducing the likelihood of stent fracture, granuloma formation, or mucus retention as observed in this study, excluding one dog that developed granuloma formation after stent fracture [69]. An alleviation of coughing after the procedure was noted, which stabilized with follow-up until day 90 [62] and was not included in the second study. In addition, it is worth mentioning the easy and quick removal of the stent where the removal time ranged from 2–12 min depending on the degree of epithelialization of the stent [69]. Given these results, it should be considered whether this type of stent would not only be a future solution for children with tracheomalacia but also for companion animals with tracheal collapse due to the lower number of complications as well as arguably lower cost of stent production than traditional cross-braided stents.

**Figure 2:**
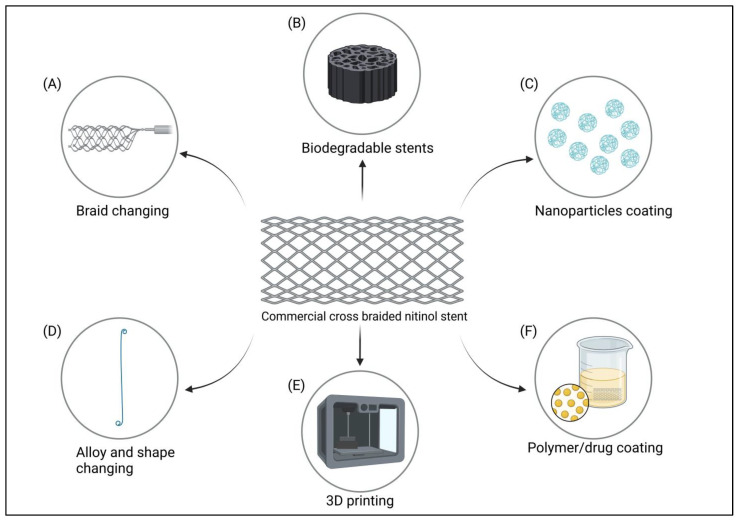
Types of stents, their coatings, and their possible use in veterinary medicine. (**A**) Replacement of typical cross-braided stents with cross-hook or other types of braided stents. (**B**) Use of biodegradable materials, allowing gradual, controlled absorption of the stent. (**C**) Use of nanoparticles with different biological properties. (**D**) Changing the material used for the stent and altering the shape to allow the stent to adapt to the target organ. (**E**) Three-dimensional printing to produce a stent with an exact planned shape, based on the printing material used. (**F**) Coating stents with polymers that serve as drug carriers, the second component of a two-component coating which allows to obtain a material with specific biological properties. 3. Stent modifications and their possible use in veterinary medicine.

The material used for ureteral stenting is mainly based on polymeric materials. Currently, double pig-tail stents are used because of their high biocompatibility and stability in the ureter due to their specific design [22,27,30,65]. Thanks to the material from which it is made, it can remain in the ureter for months, after which it can be easily removed. The use of biodegradable stents is also indicated; however, their clinical translation in veterinary medicine so far remains undescribed, as all published work has been one of the implementation steps required to introduce them to the human medicine industry [70,71,72,73]. Although such studies sometimes use a canine model [71], this does not translate to veterinary medicine due to the lack of available clinical trials. As in the case above, stents coated with various nanoparticles, polymers, or drugs are used in animals with a view for future implementation in human medicine (Figure 2). So, despite documented work in dogs using paclitaxel [71], cefotaxime [74], or sirolimus [75], these have not been found in clinical translation in veterinary medicine. To date, there has been one study in terms of stent modifications dedicated to dogs. The authors focused on describing a stable, biocompatible coating with low cytotoxicity, and high degrees of cell adhesion, which, moreover, would not induce an inflammatory reaction. For the polymers tested, such as polycaprolactone (PCL), poly-l-lactic acid (PLA), and chitosan, it was the latter that proved to be the compound that met all the assumptions, proving that it could be one of the future directions for the use of stents in veterinary medicine [76]. In addition, the interaction of chitosan with some nanoparticles such as silver nanoparticles (AgNPs) which release silver ions shows synergistic antimicrobial activity [77] which is a strictly desirable effect in the treatment of tracheal inflammation or ureteral strictures in dogs.

## 3. Stent Modifications and Its Possible Use in Veterinary Medicine

In veterinary medicine, stent modifications are not commonly used, in fact, it can be said that stent modifications are not encountered in clinical practice. The use of such solutions has been mainly scientific or performed as a necessary research step in the registration process of a new generation of stents for humans, as mentioned above. In contrast, scientific as well as clinical work on the use and suitability of stent coatings in human medicine is much more developed. In fact, classic stents are no longer used as much, having been supplanted by a new generation of drug-coated stents based on antimicrobial, anti-inflammatory [61,78], or antiproliferative properties, inhibiting granulation tissue growth [3,79]. The substances and their properties used in this article are shown in Table 1. Moreover, the ever-increasing problem of developing arteriosclerosis [80] in humans has contributed to the thriving development of vascular stenting procedures, thus forcing researchers to develop new solutions to prevent red blood cells from depositing on the stent surface, forming a clot and causing restenosis. In addition, due to the risk of neointima hypertrophy on the stent surface, certain solutions are expected to prevent blood clotting and neointima hyperproliferation [81,82]. Among the available scientific positions, there are reports whose results may inspire veterinary researchers on the improvement of previously used stents and their introduction into clinical practice.

### 3.1. Stents Modifications Related to Tracheal Collapse—Granulation Tissue Prevention

In tracheal stenting, the main complication after insertion of a tracheal stent is the formation of granulomas due to the over-reactivity of tracheal epithelial cells as well as connective tissue cells after contact with the stent [101]. This leads to restenosis and the necessity to remove the accumulated tissue. Such situations often lead to secondary bacterial infections complicating the whole process. This problem also occurs in human medicine, where some modifications and solutions have been introduced, the results of which are extremely promising and potentially applicable in veterinary medicine. So far, there has been one paper on the modification of a stent, evaluated for tracheal collapse in dogs. In vitro studies of three polymers showed that chitosan had antimicrobial properties [102], negligible cytotoxicity, prevented stent migration, and thanks to its mucoadhesive properties, adhered well to the trachea. In addition, its production costs were not high, so its use seems promising for treating tracheal collapse in dogs [76]. However, further research is needed, primarily using in vivo models. Among the available studies in the literature, there are other noteworthy solutions already tested in laboratory animals. The previously discussed anticancer drug, paclitaxel, showed good immobilization on commercially used stents which resulted in the paclitaxel loading of almost 16.5 mg/stent, which is considered a safe therapeutic dose [103]. In vivo studies comparing three types of stents showed that those coated with paclitaxel had satisfactory effects limiting granulation tissue formation in mice. Interestingly, a correlation was found between the increase in IL-8 in blood and the induction of restenosis, which may serve as a diagnostic marker after endotracheal stent placement [104]. Subsequent in vivo studies indicated a significant reduction in hyperplastic tissue compared to the control group by 5 months. Also, the concentrations of released drugs were not shown to be too high in the stent area nor in the adjacent tissues. The stent proved to be safe and biocompatible and serum paclitaxel levels were unmeasurable [105]. The curiosity and usefulness of the results are emphasized by the fact that the in vivo models were dogs. Another anticancer drug tested as drug-eluting stents (DESs) in tracheal stenosis was cisplatin with AgNPs. Again, the results showed promise. In vitro as well as in vivo tests indicated a significant reduction in hyperplastic tissue, showing high biocompatibility and good antimicrobial activity due to the addition of AgNPs [106]. Moreover, it was found to reduce the expression of IL-8, TNF-α, IL-1α, PCNA, α-SMA, and CD68 as well as inhibit collagen deposition indicating anti-hyperplastic and anti-inflammatory properties [79]. In addition, therapeutic levels of cisplatin were found at 5 weeks after the procedure [79,107]. However, the introduction of anticancer drugs as DESs, in veterinary medicine, may be associated with legal restrictions [108]. Sirolimus is a well-known and registered immunosuppressive drug used as a DES in vascular disease with high efficacy [109]. Due to the strong tissue reaction after insertion of the stent into the trachea, it was decided to test its anti-hyperplastic properties in in vivo studies in rabbits; however, 12 months after the start of the procedure, no significant differences were found between the control group with the bare metal stent in place and the study group with sirolimus [110]. Different results were obtained by the researchers when sirolimus was used as a DES during both in vitro and in vivo studies. A reduction in collagen deposition as well as fibroblast proliferation was found, which was confirmed in a mouse model [111]. The difference in results may be due to the choice of drug carrier. In the above work, two biodegradable polymeric stents were used, based on a mixture of PCL with PLA and poly(D, L-lactide-co-glycolide) (PLGA), which yielded the expected results, especially for the former one. The use of a suitable stent as a drug carrier allows for efficient and controlled release extending the duration of the therapeutic effect, thus controlling the dose of the drug [111]. In addition to the use of polymers for the stent, it is possible to coat metallic stents with a polymer coating to serve as a drug-releasing carrier [112,113,114]. This is an interesting option because, as described above, it allows for a more controlled release of the drug, additionally having biological properties. Some authors [17] studied the properties of three polymeric carriers (PLGA, PLA, and PCL) releasing methylprednisolone sodium on a nitinol stent as a scaffold. Despite the least resistance against mechanical forces as well as a relatively low degree of drug coverage (15.7%), it was the PLGA-based carrier that showed the best anti-inflammatory and antiproliferative properties, while the other polymers showed a significant degree of granulation tissue engraftment despite the high drug coverage of PLA (96.3%). In addition, significant granulation tissue activity was recorded for 10 days after tracheal stent placement, making the authors point out that this is the most critical time period after the procedure [17]. More advanced solutions like modifying 3D-printed stents by coating them with drugs are also being considered, with high efficacy in preclinical studies [16,115,116].

### 3.2. Biofilm Formation in Urinary Tract Infection Related to Stent Procedures

**Figure 3:**
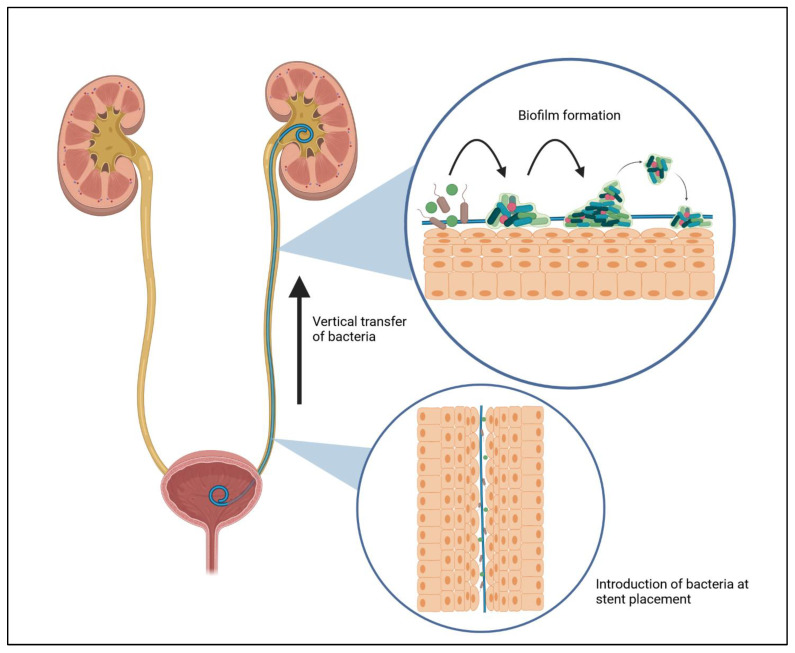
Initiation process of biofilm formation after the stent placement procedure in the urinary system.

Currently, there are many papers available that are dedicated to the modification of stents in the treatment of ureteral strictures. The indications for its insertion in human and veterinary medicine are almost the same. The presence of bacteria capable of causing infection after stent placement is determined by their introduction during the procedure or by their ability to move in the fluid. The time of stent colonization is observable two weeks after the procedure, with bacterial colonization being more aggressive the longer the stent insertion procedure takes place [117]. Other authors report that the first signs of colonization appear as early as 24 h after the procedure [118] because, after only a few minutes, the surface of the stent interacts with the host’s urine sediment, creating a substrate for bacterial adhesion [119]. Additionally, there is a higher likelihood of biofilm formation and re-obstruction of the ureteral lumen in patients with long-term stent placement [120], which in veterinary medicine affects almost every patient. Consequently, strategies in the context of prevention of its formation and control may be crucial. A biofilm is a tightly organized bacterial formation, the products of which form a cohesive, hermetic entity capable of spreading and increasing in size (Figure 3). It is estimated that a substantial percentage, roughly 50–80%, of bacteria possess the capability to create biofilms [121,122]. Its formation can be divided into three phases, the first of which is reversible and removable by conventional methods. The stent placed in the urinary outflow tract becomes a potential substrate for the adhesion of various components including polysaccharides, proteins, or glycoproteins found in the urine sediment. This becomes the primary medium for planktonic cells initiating the formation of the future biofilm [123]. Proliferating bacterial cells as well as those deposited de novo begin to produce exopolymeric substances which start the cross-linking process. This phenomenon begins the second irreversible phase of biofilm formation. Its maturation and the formation of an extracellular matrix capable of retaining nutrients and capturing interbacterial communication signals (quorum sensing, QS) completes the third phase, and the biofilm thus formed is capable of expanding in size and spreading. A cluster of such a large and diverse bacterial colony increases its resistance to antibiotics by more than a thousandfold, making therapy with pharmaceuticals practically impossible [15,124]. For this reason, it was decided to look for solutions related to stent modification and counteracting biofilm formation [59,78]. This strategy distinguishes three mechanisms of action on which stent modification is based. The first works are based on the principle of biomechanical precipitation of bacterial particles thanks to the special design of the stent net, through which biofilm removal becomes achievable. The second, on the other hand, works based on low-adhesion surfaces, which, thanks to their superhydrophilic or superhydrophobic properties, prevent bacteria from adhering to the stent surface [125]. However, their use in veterinary medicine seems a distant thing, so the most important and third modification of the stent is to coat it with various compounds with biocidal properties.

### 3.3. Strategies for Biofilm Prevention in Stent Designed for Ureter Obstructions

The mechanism of antimicrobial action will vary depending on the biologically active substance immobilized on the surface of the stent. The best-known and broadest group of antimicrobial agents are antibiotics, whose molecules can also be deposited on the stent surface. Moreover, their efficacy has been confirmed in several studies, showing strong biocidal properties for ureteral stents [78]. However, the high antibiotic resistance of biofilm-forming microcolonies and the limitation of antibiotic use prompts researchers to search for alternative pathways to combat microorganisms. One solution may be the use of antimicrobial peptides. Their cationic structure leads to non-specific interactions with exopolysaccharides present on the bacterial surface permeabilizing the cell membrane and disrupting intracellular function, including inhibition of DNA or RNA synthesis [89,90]. One of these is tachyplesin III, isolated from horseshoe crabs. As it turns out, its broad spectrum of action against Gram-negative as well as Gram-positive bacteria can inhibit bacterial growth up to 1000 times. It also shows no signs of cytotoxicity, which supports its safe use [126]. In addition, its efficacy has been found against multidrug-resistant strains of *Pseudomonas aeruginosa* and *Acinetobacter baumanii*, one of the more commonly cited urinary biofilm isolates [127,128]. Recently published studies have identified a mussel-inspired clickable antibacterial peptide with high biocompatibility, antimicrobial activity, as well as anti-incrustation properties [129,130]. Such solutions seem to be promising, but the high cost of production, regulatory restrictions, as well as bioengineering capabilities seem to preclude their application in veterinary medicine. In addition to these, there are several other solutions that seem to be rather utopian thoughts in veterinary medicine, and their introduction will be directed towards human medicine. Among them, we can mention coatings based on heparin [131], bacteriophages, or enzymes that digest the bacterial wall [78].

There are some modifications in ureteral stenting, the use of which in veterinary medicine can bring many benefits from a clinical point of view, with not so much financial burden for the owner. Namely, those are drug-eluting stents, but also polymers used as their carriers. One of them is triclosan, a representative of the chlorinated phenol group, whose first in vitro studies showed positive benefits, retaining its antimicrobial activity 3 months after immobilization on the surface of the stent [10], which was confirmed by later studies suggesting that it could be a good antimicrobial agent treated for ureteral stents, excluding *Pseudomonas aeruginosa*, which has shown significant resistance [132]. In vivo studies in rabbits confirmed earlier reports showing high efficacy against artificially induced *Proteus mirabilis* infection [133]. However, later studies on groups of patients showed that triclosan did not significantly reduce the number of isolated bacteria in the control group but favorably reduced other symptoms, albeit those related to pain [134,135]. Promising results have been obtained by researchers from the use of zotarolimus, a drug in the immunosuppressant group. In vivo studies on porcine and rabbit ureters showed a significant difference in stent patency, where all the ureters were unobstructed in the study groups, but with slight hyperplasia, in contrast to the control group, where the majority of the ureters showed significant stent encrustation and obstruction [136]. Despite the promising results, it was not decided to continue the study. Indomethacin was another agent considered for coating stents dedicated to the urinary tract. Preclinical studies admittedly showed efficacy by reducing stent encrustation and decreasing the inflammatory response, but further studies were not continued [137,138,139,140]. Paclitaxel is a well-known anticancer drug that has long been known and approved as a drug-eluting stent for coronary artery stenting in humans. Its action is based on disrupting the balance between polymerization and depolymerization of microtubules, thus disrupting cytoskeletal function leading to cell death [83]. The first studies on urinary tract stenting were specifically in dogs, although not in the ureters, but in the urethra, indicating a reduction in hyperplasia of the surrounding tissue [141]. Similar results were obtained by researchers on the urethra of rabbits thus showing a reduction in hyperplastic tissue, inflammation as well as scar formation compared to control groups [142]. The use of paclitaxel-coated stents in the ureteral area also provided measurable benefits, reducing inflammatory reactions while maintaining ureteral patency in contrast to the control group where some of the ureters were obstructed [143]. In addition, the high healing efficiency of anastomoses with low urothelial tissue reaction in a paclitaxel-coated stent after artificially induced ureteral reconstruction in the rat was indicated. Good healing of the anastomotic site, reduced inflammatory reaction, as well as reduced urothelial hyperplasia, were observed [144]. However, for ureter stenting, despite the promising results, it should be kept in mind that paclitaxel is a cancer drug, which conditions legal restrictions on its use in non-cancerous cases in animals, making it impossible to register it in certain countries.

Taking into account the above reports, it can be said that there are many solutions that would be of great benefit if introduced into veterinary medicine, but further clinical studies using companion animals affected by tracheal collapse or ureteral obstruction are needed to confirm studies on laboratory models.

## 4. Novel Nanotech-Based Coatings for a Better Tomorrow

Solutions in veterinary medicine based on nanotechnology are highly developed and widely used. Examples of the use of nanotechnology can be noted in such veterinary fields as diagnostics, treatment, nano-vaccines, nano-adjuvants, nutrition, and reproduction [145,146]. Moreover, there are known in vitro reports using titanium plates coated with titanium nanotubes, additionally loaded with AgNPs for veterinary implants to support bone regeneration [147]. Another study, on six canine patients, showed faster bone regeneration and reduced the inflammatory process after tibial plateau osteotomy [148]. However, other than these, nanoparticle-coated medical devices in veterinary medicine are rather rare. Nevertheless, many of them have very interesting antimicrobial or antiproliferative properties, making them a useful tool to counter complications that arise after stent placement. In addition, it is possible to combine them with various polymers serving as drug carriers that also have their biologically active properties, making it possible to obtain a coating with broad properties [77,78].

The most widely used nanomaterial with antimicrobial properties for medical devices is undoubtedly silver due to its high antimicrobial toxicity and low host cell cytotoxicity, as widely documented in the literature [149]. However, its use requires carefully performed and customized coating procedures due to its variable biological and cytotoxic properties dependent on size, shape, and concentration [150]. In addition, interactions between the polymer carrier and that incorporated in its solution AgNPs affect its release and thus antimicrobial activity [151]. The chitosan–AgNP biopolymer composition exhibits a strong antimicrobial response to inhibit biofilm formation, but the antimicrobial effect varies depending on the type of nanosilver [77]. It was shown that bifunctional polymer nanocomposites of another chitin derivative with AgNPs exhibited high antimicrobial activity, including against a methicillin-resistant strain of *Staphylococcus aureus* (MRSA), as well as antifungal activity in both in vitro and in vivo studies on a mouse model [152]. In addition, greater antimicrobial activity was achieved by using polyvinyl alcohol (PVA) as a carrier for AgNPs due to its faster release [151]. It was also indicated that the nanocomposite coating consisting of AgNPs and polytetrafluoroethylene (PTFE) significantly inhibited bacterial migration and biofilm formation, and prevented encrustation [153], which may prove to be a useful solution in ureteral stenting. In addition, the effectiveness of AgNPs has been demonstrated in their combination with cisplatin as a means of preventing granuloma formation and secondary bacterial infections [79,124]. The use of materials based on graphene and its derivatives with AgNPs indicates their possible use as effective coatings during stenting [78]. In addition, graphene and its derivatives themselves show interesting biological properties. Currently, the possibilities of its application in human medicine are being explored for orthopaedic, neurological, or cardiovascular implants [154]. Its unique structure allows for antimicrobial properties, and in combination with certain drugs and polymers, it shows antiproliferative activity [155,156] which could serve as a potential application in tracheal collapse in veterinary patients.

## 5. Limitations

Our review focuses on addressing the main issues related to stenting in veterinary medicine. In the sense of stenting the urethra or gastrointestinal tract, it is said to be more of a palliative treatment [23,53]; therefore, stent modifications in this regard would not be a necessity. However, significant postoperative complications in the context of tracheal and ureteral stenting remain a major concern. The same challenges are being addressed in human medicine, which is why there has been a lot of work in this area to date.

The main limitation of this review was the nature of the studies conducted because all of the aforementioned studies were conducted with an eye toward implementation in human medicine, except for one in which researchers evaluated various polymeric substrates and their behavior in vitro as a possible coating to be used during tracheal stenting in dogs [76]. Because research has not been continued, and previous work based on evaluating the biological properties of stent coatings has focused on human medicine, there are currently no reports in the literature of clinical applications of these solutions in pets. Admittedly, there are papers available in which the laboratory model was a dog in the case of tracheal stenting. Despite the very promising results, it must be emphasized that these were healthy dogs, maintained under laboratory conditions. The most common patients with tracheal collapse are small dogs, usually with an overactive or even choleric temperament, which translates into their high reactivity exposing the trachea to mechanical stimulation which certainly affects the behavior manner of the stent in its lumen. Given this, laboratory conditions certainly do not reflect the daily environment and the various types of stimulation the animal encounters. In addition, the use of anticancer drugs in such situations could face some legal restrictions [105,108].

Ureteral stenting faces a similar problem. There are no papers based on retrospective cases describing clinical outcomes from stenting ureters coated with various compounds. In vivo studies tested on canine models, despite the promising results, in this case were also conducted under laboratory conditions that differ from the daily use of the animal [141]. Therefore, despite many reports of the coatings used to coat stents, both in vitro and in vivo, many clinical studies are needed, with long-term observations that will convey the full-scale utility of the coatings used.

## 6. Conclusions and Future Perspectives

(1) This review brings together information on the most common conditions treated with stenting procedures in veterinary medicine. Despite promising results shortly after the procedure, serious post-surgical complications occur over time, forcing owners to undertake further surgical measures. Undoubtedly, this is a major economic expense for the owner as well as another interference with the animal’s body, which significantly affects its welfare. So far, no commercial solutions have been introduced to effectively counteract the excessive growth of granulation tissue in the case of tracheal collapse, or bacterial infections after placing a ureteral stent on the veterinary market.
(2) Currently, the most commonly used material in the production of stents in veterinary medicine is nitinol with a cross-braided stent. However, solutions have emerged that modify the design as well as the method of stent implantation, which has translated into a much better postoperative outcome compared to commercially used stents [68,69]. However, they are not available for widespread use, but it seems that the use of such solutions may be justified because they do not need additional procedures related to stent modification, which will also reduce production costs. This is one option that may find viable application in veterinary medicine.
(3) Compared to human medicine, veterinarians do not have available stents that fit into the DES category using coatings of polymers, drugs, nanoparticles, or antimicrobial peptides. Nevertheless, with the available literature focusing on the study of new coatings using in vitro testing, we can determine the future direction of stenting research in veterinary medicine. In addition, there are many reports using in vivo animal models. Through animal studies, we can confirm high efficacy against granulation tissue hyperresponsiveness through the use of cytostatics such as paclitaxel and cisplatin, as well as compounds that inhibit cell proliferation—sirolimus. In addition, antimicrobial compounds based on antimicrobial peptides, immunomodulatory drugs, and in particular, nanoparticles, effectively inhibited bacterial growth and biofilm formation.
(4) In consideration of the above, prospects should focus on implementing new solutions related to surface modification of applied stents. Multicomponent coatings containing both antiproliferative and antimicrobial compounds seem to play a crucial role [79]. Their synergistic action allows for effective protection and reduction of postoperative complications. In addition, stent modifications that change their shape or braided nature also seem to be a good option. A different distribution of the stent surface forces about the surrounding tissue, allowed the tracheal epithelial cells to adapt better, reducing the negative effects after stent placement [68,69]. It should be noted that most of the studies were scientific, where the animal models were mice, rabbits, pigs, and even healthy dogs. However, there are no studies relating to veterinary patients with real-world clinical problems such as tracheal collapse or ureteral obstruction using modified stents. In addition to developing new solutions, there is a need for clinical trials using veterinary patients.

## Figures and Tables

**Table 1:** Materials listed in review as possible biological coatings for veterinary stents.

Properties	Substances	Mechanism of Action	Author
Antiproliferative	Paclitaxel	Disrupting the balance between polymerization and depolymerization of microtubules, thus disrupting cytoskeletal function leading to cell death	[83]
	Cisplatin	Crosslinking with the purine bases, which interferes with DNA repair, causing damage	[84]
	Sirolimus	Complex sirolimus-FKBP12 acts as an inhibitor of kinase mTOR which finally results in the blocking of the cellular pathway at the junction of G1 and S-phase phase	[85]
	Zotarolius	Inhibition of mTOR kinase by the complex of zotarolimus-FKBP12 leading to inhibition of cell proliferation	[86]
Antibacterial	Cefotaxyme	Inhibition of bacterial wall synthesis	[87]
	Tachyplesin III	Inhibition of FabG enzyme activity and blocks NADPH bindings of bacteria	[88]
	Antibacterial peptides	Permeabilizing cell membrane and disrupting intracellular function	[89,90]
	Triclosan	Inhibition of bacterial fatty acid synthesis	[91]
	AgNPs	Penetration of cell membranes by nanoparticles leads to the production of reactive oxygen species leading to the disruption of bacterial cell signaling pathways	[92]
	Graphene	Free edges of GR sheets containingcarboxyl groups provide an attachment site for bacteria by disrupting the integrity of their membranes	[93]
	Graphene oxidate	Disruption of phospholipid integrity leads to bacteria membrane damage, the generation of reactive oxygen species causing DNA fragmentation and cell death	[93,94]
Polymers	Chitosan	Drug carrier, controlled and targeted release of drug in different formulations	[95]
	Polycaprolactone (PCL)	Drug carrier, controlled and targeted release, enhanced properties of used drugs	[96]
	Poly-l-lactic acid (PLA)	Drug carrier for a variety of substances in different formulations, controlled and targeted release	[97]
	Poly(D, L-lactide-co-glycolide) (PLGA)	Controllable drug release profile, ability to alter surface with targeting agents for diagnosis and treatment	[98]
	Polyvinyl alcohol (PVA)	Drug sustained release carrier, targeted releasing at specific sites of the body, controlled rate of drug release	[99]
	Polytetrafluoroethylene (PTFE)	Drug carrier	[100]

## Data Availability

Not applicable.
